# Genotyping of ESBL Producing Uropathogenic *Escherichia coli* in West of Iran

**DOI:** 10.1155/2014/276941

**Published:** 2014-04-15

**Authors:** Parviz Mohajeri, Gita Darfarin, Abbas Farahani

**Affiliations:** ^1^Department of Microbiology, School of Medicine, Kermanshah University of Medical Sciences, Kermanshah 67148-69914, Iran; ^2^Department of Microbiology, Ayatollah Amoli Branch, Islamic Azad University, P.O. Box 678, Amol, Iran; ^3^Student Research Committee, Kermanshah University of Medical Sciences, Kermanshah 67148-69914, Iran

## Abstract

*Background and Objective.* Urinary tract infection (UTI) is one of the most common bacterial infections in the world. Molecular fingerprinting of UTI isolates such as pulsed-Field Gel Electrophoresis using for Clonal distribution and determine of predominant type. The aim of the study was to determine genotyping of ESBL producing UPECs. *Material and Methods.* 200 UPEC isolates from outpatients with UTI were obtained. Antimicrobial susceptibility and interpretation were performed by disk diffusion. Virulence factors for UPECs were screened by using PCR. UPECs were analyzed by Pulsed-Field Gel Electrophoresis and images analyzed by Phoretix1DPro software. * Results.* A total of 200 isolates of UPECs, 24.5% (*n* = 49) of isolates, were positive for ESBL production. Resistance ranged from 0% for amikacin and imipenem to over 93.9% for carbenicillin and ampicillin. Frequencies of haemagglutination, haemolysin, and hydrophobicity were 51%, 18.3%, and 14.28%, respectively. A total of 10 different genotypes were obtained, which include nine common clones and one single clone. * Conclusion.* We confirmed the prevalence of virulence phenotyping especially Haemagglutination among UPEC strains and that it can also contribute to virulence in these strains. Large diversity in genotypes was observed in the isolates that could be indicative of different sources of infection in community acquired.

## 1. Introduction

Urinary tract infection (UTI) is one of the most common bacterial infections in various parts of the world and also UTIs may be an emerging problem in patient in different parts of the world with high medical costs [1]. Bacterial adherence to uroepithelial cells is essential for the initiation of infection in UTI [2].* Uropathogenic Escherichia coli *(UPEC) is a normal flora of the human intestinal, although UTI isolates have acquired specific virulence attributes, which confer the ability to adapt these isolates to new location and then cause a broad spectrum of disease [3]. UPEC isolates are genetically heterogeneous group of various virulence factors associated with colonization and survival of bacteria in the urinary tract [4]. UPEC strains are able to produce various types of adhesions necessary for attaching to receptors along uroepithelial cells including P fimbriae (coded by pap), S fimbriae (coded by* sfa* genes), and Afa adhesions (coded by* afa* genes) [5, 6]. Adhesion factors of the bacteria to bind to the surface, creating colonies, invasion, and proliferation of bacteria in the urinary tract epithelial cells have an important role [7]. Other characteristics are also important for the UPEC ability to cause UTI such as cell surface. Hydrophobicity, Hemagglutination, and hemolysis erythrocyte of virulence factors of* Escherichia coli* strains causing extraintestinal infections in humans are considered. These factors cause tissue damage, facilitating bacteria release, releasing of host nutrients, and spread to other parts of the body [8, 9]. Cephalosporin's belonging to commonly used for treatment of infections by* Escherichia coli* in UTI. However, resistance to these antibiotics is increasing in last decades [10]. This has been attributed to emergence of strains that can be producing extended-spectrum *β*-lactamases (ESBLs) [11]. UTIs are the most common types of community associated ESBL infections caused by* E. coli*. Therefore, ESBLs producing* E. coli* from UTIs are a feasible bacterial population for a comparative study [12]. Molecular fingerprinting of bacterial isolates for their clonal distribution and determination predominant type of isolates are carried out by means of various molecular techniques. In recent years Pulsed-Field Gel Electrophoresis (PFGE) method is the most successful fingerprinting technique for molecular epidemiology purposes and it has been used for the most common pathogen range of bacterial species [13, 14].

The aim of the study was to determine genotyping of ESBL Producing UPECs and distribution of virulence factors in difference pulsotypes of these isolates by using Pulsed-Field Gel Electrophoresis method and finally we report evaluation pulsotypes in western of Iran.

## 2. Materials and Methods

### 2.1. Bacterial Isolates

In total, 200 UPEC isolates from outpatients with UTI were collected in the community in west of Iran from February 2012 to February 2013. The diagnosis of UTI isolates was clinical presentation with typical clinical symptoms of lower urinary tract symptoms such as dysuria, fever, frequency, urgency, and growth >10^5^ colony (10^5^ cfu)* E. coli *per mL in urine sample. All isolates were identified by biochemical and isolates were confirmed using API20E kit. In our study only the patients with community acquired UTI were included and patients with nosocomial infections which were defined as infections that were noted 48 h or 72 h after admission discharge [15].

### 2.2. Susceptibility Testing and ESBL Detection

Antimicrobial susceptibility was performed by disk diffusion method according to Clinical Laboratory Standard Institute (CLSI) [16]. The antibiotics used were as follows: Cefoperazone (30 *μ*g), ceftriaxone (30 *μ*g), cefuroxim (30 *μ*g), Ciprofloxacin (5 *μ*g), piperacillin (100 *μ*g), gentamicin (10 *μ*g), amikacin (30 *μ*g), imipenem (10 *μ*g), ampicillin (10 mg), aztreonam (10 mg), trimethoprim-sulfamethoxazole (30 *μ*g), Carbenicillin (50 *μ*g), nitrofurantoin (300 *μ*g), and ofloxacin (5 *μ*g) (MAST, Merseyside, UK). Disks containing 30 *μ*g of ceftazidime, ceftriaxone, cefotaxime, and cefepime plus 10 *μ*g of clavulanic acid were tested on a Mueller-Hinton plate for detection of ESBL producing bacteria. A positive test result was defined as increase of more than 5 mm in zone diameter compared with a disk without clavulanic acid [17].

### 2.3. PCR Assay

Virulence factors for UPECs were screened for three main groups of genes using PCR:* pap*,* afa,* and* sfa* genes [4].

Haemolysin, haemagglutination, and hydrophobicity tests were performed according to the method described elsewhere [18, 19]. 

**Figure 1:**
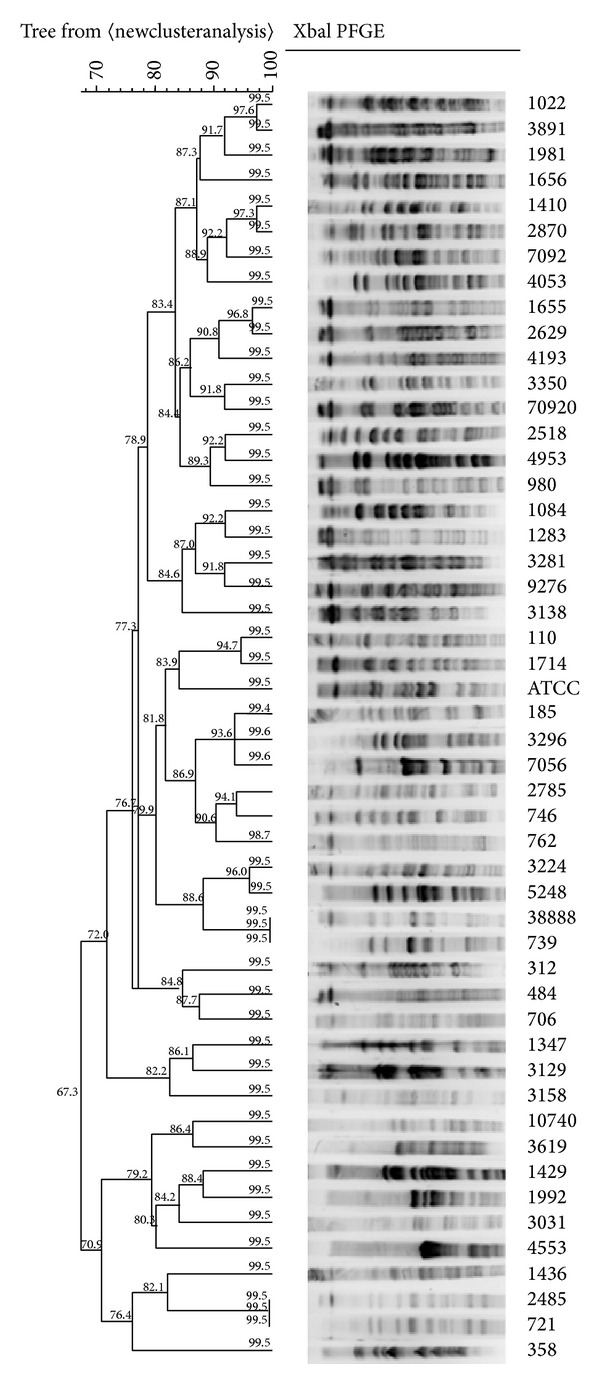
Pulsed-field gel electrophoresis dendrogram of UPEC isolates. The vertical black line shows the* 80% cut-off.*

**Figure 2:**
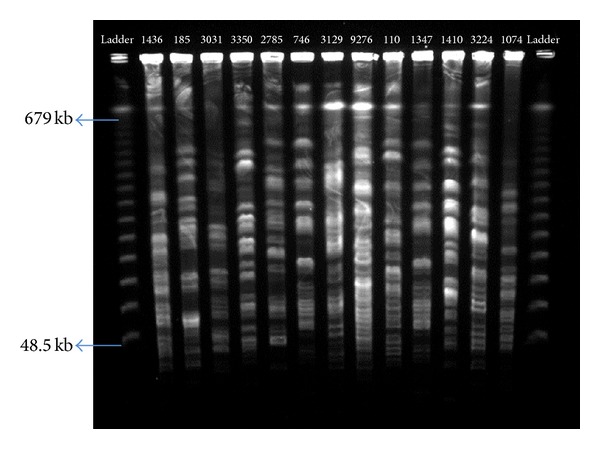
CHEF profiles of UPEC strains isolated. Lateral lanes contain Lambda Ladder PFG Marker. Size range: 48.5 kb to 679 kb (14 fragments).

*Pulsed-Field Gel Electrophoresis*. The isolates were analyzed by PFGE using methods as described by Mohajeri et al. [20]. We used ATCC 25922 for external reference. Genotyping of all organisms was performed with* XbaI* (Fermentas) digestion. Electrophoresis in a pulsed-field electrophoresis system (Chef Mapper; Bio-Rad Laboratories, Hercules, CA, USA) by program two states with conditions: temperature 14°C; voltage 6 V/cm; switch angle, 120°; switch ramp 2.2–54.2 s for 19 h. The gels were stained with ethidium bromide and patterns were photographed with UV gel Doc (BIO-RAD, USA) (Figure 1). The Lambda Ladder PFG Marker (NEB, US) was used as the standard/reference strain. A total of 14 bands were found in the standard/reference strain ranging in size from 679 to 48.5 kb (Figure 2). Analysis of banding patterns was done with the Molecular Analyzed Phoretix1DPro software (Total Lab, Newcastle upon Tyne, UK). All dendrograms were obtained to relatedness of pulsotypes for all UPECs isolates, and cut-off levels of 67.3 and 99.5% were applied to this dendrogram. With the 80% cut-off, UPECs isolates differing by up to seven DNA fragments were clustered together in this study (Figure 1); the PFGE DNA patterns obtained were interpreted and compared as described by Tenover et al. [21].

### 2.4. Statistical Analysis

A statistical comparison of the frequencies of Pulsotypes presence in UPECs isolates was conducted by chi-squared test (variables were analyzed by chi-square test). All *P* values were two-sided with a *P* value of < 0.05 considered to indicate significance.

## 3. Result

A total of 200 isolates of UPECs were collected from hospitals in Kermanshah (Iran) that all isolates obtained from outpatients. Double disc synergy test (DDST) methods showed that 24.5% (*n* = 49) of isolates were positive for ESBL production. We evaluated 49 patients (9 male and 40 female), a female-to-male ratio of 4.4 : 1. The majority of UTI isolates were obtained from adult withmean age for male 32.76 ± 23.31 and mean age for female 28.23 ± 27.04. The rates of resistance ranged from 0% for amikacin and imipenem to over 93.9% for carbenicillin and ampicillin (Table 1). Frequencies of haemagglutination, haemolysin, and hydrophobicity were 25 (51%), 9 (18.3%), and 7 (14.28%), respectively (see Table 3). Of UPEC strains in this study, a total of 10 different genotypes were obtained, which include nine common clones and 1 single clone. Of nine common types observed, type A (with 16 isolate) was the predominant type. That type as B, C, D, E, F, G, H, I, and J was named, respectively, each with 8, 5, 4, 4, 3, 3, 3, 2, and 1 isolate were included.

More resistance was observed in A, B, C, D, E, F, and G Pulsotypes, respectively. Virulence phenotyping more Frequencies in pulsotypes: A, B and C. More details are given in Table 2. Distribution of* afa*,* pap,* and* sfa *in total isolates, 9 (18.9%) for each gene and in pulsotypes A (16 member): 4 (25%), 3 (18.75%), and 1 (6.25%), respectively (see Table 3).

## 4. Discussion 

UTIs are regarded as a health problem around deferent regions of the world. These isolates are risk for public health such as in both inpatient and outpatient specially in some regions of Iran [22]. Our results show that 49 were positive for ESBL production and these isolates had significant resistance to Carbenicillin, Ampicillin, Cefuroxim, Trimetoprim-sulfamethoxazole, and Piperacillin, respectively. Adhesion factors (or Virulence factors) of the bacteria to bind to the surface, creating colonies, invasion, and proliferation of bacteria in the urinary tract epithelial cells have an important role. Our results show that high frequency of Haemagglutination (51.02%) Haemolysin (18.36%) in ESBL producing UPEC strains, those were higher than to other virulence factors. PFGE analysis has been used in epidemiological and molecular studies of numerous bacterial and is gold standard for molecular epidemiologic in many bacteria such as* E. coli* isolates. This technique is better to identify the source of infection and spread [23]. In the present study, the high similarity (96–99.5%) between isolates, indicating that the prevalence of these isolates among the community. In study of Anvarinejad et al., the lowest similarity was observed and failed to find an association between spread and colonization of UTI isolates [24]. More Virulence factors present in pulsotype A. The presence of these isolates with adhesion factors (such as* pap*, * sfa*, and * afa*) in this pulsotypes may be spread mostly among people in the community. In this study, we confirmed the prevalence of virulence phenotyping especially Haemagglutination (A significant association between production of mannose-resistant haemagglutination and Haemagglutination observed, *P* < 0.001) among UPEC strains (partially in pulsotypes A and B) and that it can also contribute to virulence in UPEC strains. We guess virulence factors have been proposed to play a role in the induction of outbreak of pulsotypes A, B, C, and D in future. The present study revealed that resistant rates to available antibiotics are very high. It seems that infection control strategies may help to control the evolving problem of UTI infections and prevent an epidemic of life-threatening community acquired infections.

## 5. Conclusion

In our study, we found that the antibiotics ampicillin, carbenicillin, and piperacillin are not as a choice for treatment of UTI patients. Overall, our study indicated that Uropathogenic* Escherichia coli* strains are spreading and urinary tract infection (UTI) is one of the most common bacterial infections in western Iran community. Note that the similarity between some of the isolates was 100%. The high similarity could be a warning to the community. This similarity is alarm for community health and hospitals. In our study, large diversity in genotypes was observed in the isolates that could be indicative of different sources of infection in community acquired isolates. The results of this study suggest that the risk of an outbreak in the future and a larger study is needed to investigate further.

## Figures and Tables

**Table 1:** Antimicrobial-susceptibility for UPEC strains.

Antibiotics	Susceptibility; number (%) of isolates:					
	Susceptible		Intermediate		Resistant	
	Number	%	Number	%	Number	%
Amikacin	46	93/9	3	6/1	0	0
Ampicillin	3	6/1	0	0	46	93/9
Aztreonam	11	22/4	2	4/1	36	73/5
Carbenicillin	3	6/1	0	0	46	93/9
Cefoperazone	9	18/4	4	8/2	36	73/5
Ceftriaxone	11	22/4	2	4/1	36	73/5
Cefuroxim	10	20/4	1	2	38	77/6
Ciprofloxacin	26	53/1	2	4/1	21	42/9
Trimethoprim-sulfamethoxazole	12	24/5	0	0	37	75/5
Gentamicin	34	69/4	0	0	15	30/6
Imipenem	49	100	0	0	0	0
Nitrofurantoin	47	95/9	0	0	2	4/1
Ofloxacin	28	57/1	0	0	21	42/9
Piperacillin	4	8/2	0	0	45	91/8

**Table 2:** Distribution of virulence phenotyping in pulsotypes.

Virulence phenotyping	Haemolysin		Hydrophobicity		Haemagglutination		Total isolates in pulsotypes
Pulsotypes	Number	%	Number	%	Number	**%**	
A	3	18.7	3	18.7	7	43.7	**16**
B	3	37.5	0	0	3	37.5	**8**
C	2	40	2	40	3	60	**5**
D	0	0	0	0	1	25	**4**
E	0	0	0	0	3	75	**4**
F	0	0	1	33.3	2	66.6	**3**
G	0	0	0	0	2	66.6	**3**
H	0	0	0	0	2	66.6	**3**
I	1	50	1	50	1	50	**2**
J	0	0	0	0	1	100	**1**

Total isolates	9	18.36	7	14.28	25	51.02	49

**Table 3:** Distribution of virulence genes in pulsotypes.

Virulence factors	Pap		afa		sfa		Total isolates in pulsotypes
Pulsotypes	Number	%	Number	%	Number	%	
A	3	18.75	4	25	1	6.25	**16**
B	1	12.5	1	12.5	2	25	**8**
C	1	20	1	20	2	40	**5**
D	1	25	1	25	1	25	**4**
E	1	25	0	0	1	25	**4**
F	0	0	0	0	0	0	**3**
G	0	0	1	33.33	0	0	**3**
H	1	33.33	0	0	0	0	**3**
I	1	50	1	50	1	50	**2**
J	0	0	0	0	1	100	**1**

Total isolates	9	18.36	9	18.36	9	18.36	49
